# Differential Nervous Necrosis Virus (NNV) Replication in Five Putative Susceptible Cell Lines

**DOI:** 10.3390/pathogens10121565

**Published:** 2021-11-30

**Authors:** Yulema Valero, Carmen López-Vázquez, Sandra Souto, José G. Olveira, Alberto Cuesta, Isabel Bandín

**Affiliations:** 1 Instituto de Acuicultura, Departament of Microbiology and Parasitology, Universidade de Santiago de Compostela, 15782 Santiago de Compostela, Spain; yulema.valero@usc.es (Y.V.); mdelcarmen.lopez.vazquez@usc.es (C.L.-V.); sandra.souto@usc.es (S.S.); jose.olveira@usc.es (J.G.O.); 2 Immunobiology for Aquaculture Group, Department of Cell Biology and Histology, Facultad de Biología, Campus Regional de Excelencia Internacional “Campus Mare Nostrum”, Universidad de Murcia, 30100 Murcia, Spain; alcuesta@um.es

**Keywords:** nervous necrosis virus (NNV), adsorption, replication, fish cell lines

## Abstract

Viral encephalopathy and retinopathy caused by nervous necrosis virus (NNV), is one of the most threatening viral diseases affecting marine fish worldwide. In vitro propagation of NNV strains is essential for the design of effective control measures. In the present study we analysed both the susceptibility and the permissiveness of five fish cell lines (E-11, GF-1, SAF-1, DLB-1, and SaB-1) to three NNV strains (one RGNNV, one SJNNV, and one reassortant RGNNV/SJNNV). E-11 and DLB-1 were demonstrated to be highly susceptible to NNV strains, with average adsorption efficiency (AE) values higher than 90%. SAF-1 also showed high susceptibility (AE 88%), whereas GF-1 can be regarded as moderately susceptible (AE around 50%). On the contrary, SaB-1 can be considered a poorly susceptible cell line (AE values below 20%). E-11 and GF-1 cell lines provided the highest production rates for RGNNV and RG/SJ (around 10^3^) and both cell lines can be regarded as fully permissive for these viral types. However, the SJNNV production rate in GF-1 was only 17.8 and therefore this cell line should be considered semi-permissive for this genotype. In SAF-1 cells, moderate viral replication was recorded but differences in intracellular and extracellular production suggest that viral progeny was not efficiently released. In DLB-1 and SaB-1 the final viral titres obtained in E-11 were lower than those of the inoculum. However, RNA1 synthesis values seem to indicate that RGNNV replication in DLB-1 and SAF-1 could have been underestimated, probably due to a poor adaptation of the virus grown in these cell lines to E-11. Based on all these results, E-11 seems to be the most appropriate cell for in vitro culture of RGNNV, SJNNV, and reassortant strains.

## 1. Introduction

In recent years, the spread of fish viral pathogens has increased substantially, provoking health problems and important economic losses in the fish farming industry. Among these pathogens, nervous necrosis virus (NNV) is the causative agent of the disease known as viral nervous necrosis (VNN) or viral encephalopathy and retinopathy (VER), a lethal neuropathological condition that affects fish all around the world. Typical disease signs include abnormal swimming behavior, loss of appetite, swim bladder hyperinflation, or coloration abnormalities (pale or dark), depending on the fish species, biological stage, phase of the disease, and temperature. VER-infected fish can transmit the virus to healthy fish either horizontally, fish to fish or through the water column, or vertically. Although horizontal transmission in farming sites commonly occurs among fish belonging to the same species, interspecies transmission has also been recorded [1]. NNV is a member of the genus *Betanodavirus* (family *Nodaviridae*) which comprises small (around 30 nm), non-enveloped icosahedral viruses with bipartite, positive-sense RNA genomes. RNA1 (3.1 kb) controls the synthesis of the RNA-dependent RNA polymerase (RdRp) and RNA2 codes for the capsid protein (CP) [2]. During virus replication, a subgenomic RNA3 is transcribed from the 3′ end region of RNA1. RNA 3 is not packaged into the virion and encodes the non-structural proteins B1 and B2, with regulatory functions [3,4,5,6,7,8].

Betanodaviruses are classified into four genotypes: Barfin flounder-, Striped jack-, Red spotted grouper- and Tiger puffer nervous necrosis virus (BFNNV, SJNNV, RGNNV, and TPNNV, respectively) on the basis of a variable sequence of RNA2, known as T4 region [9]. To date, RGNNV is predominant worldwide, with grouper (*Epinephelus* sp) and European sea bass *(Dicentrarchus labrax*) representing the most susceptible species to this genotype (for review see [1]). However, the emergence of reassortant strains between RGNNV and SJNNV genotypes in Southern Europe threatens Senegalese sole (*Solea senegalensis*) and gilthead seabream (*Sparus aurata*) farming [10,11,12].

The availability of both susceptible and permissive cell lines is determinant for viral isolation and for a comprehensive study of any viral agent. The lack of cell cultures that supported NNV growth in vitro represented a major constraint for its characterization for several years after the appearance of VER [13,14,15,16,17,18]. Finally, an NNV strain was first successfully isolated from diseased sea bass using SSN-1 cells derived from striped snakehead fish (*Ophicephalus striatus*) [19]. Subsequently, this cell line was demonstrated to support the growth of the four NNV genotypes [20]. Afterwards, a clone of SSN-1 cells, E 11, was reported to be more appropriate than the original cell line for the qualitative and quantitative analyses of the NNV genotypes [21] and it has been extensively used for both the isolation and culture of viral strains (for review see [1]). Almost simultaneously, the GF-1 cell line derived from the grouper (*Epinephelus coioides*) fin was shown to be susceptible to greasy grouper nervous necrosis virus (GGNNV) [22]. In recent years the number of cell lines reported to be susceptible to NNV has increased considerably (for review see [1]), although most of them have only been tested for the RGNNV genotype. In the present study we analysed the capacity to support the NNV replication cycle in five fish cell lines: E-11 and GF-1 (already described), SAF-1 derived from gilthead sea bream fin [23], and DLB-1 and SaB-1 obtained from the brain of European sea bass and gilthead sea bream, respectively [24,25]. For that purpose, the adsorption capacity and production of viral progeny (both intracellular and extracellular) of three NNV strains (one RGNNV, one SJNNV, and one reassortant RGNNV/SJNNV isolate) were tested in each of the five cell lines.

## 2. Results

### 2.1. Differential Susceptibility in the Five Fish Cell Lines

The three strains showed significant differences in the adsorption efficacy (AE) to E-11, GF-1 and SAF-1 cells (*p* < 0.0001), whereas in DLB differences were observed only between the SJNNV and RG/SJ strains (*p* = 0.033) and very similar values were recorded in SaB-1 cells. In addition, noticeable differences were seen between the adsorption values recorded in the five cell lines. To this regard, the highest AE was recorded for SJNNV strain (98.63%) in SAF-1 followed by those observed in E-11 and DLB-1 (97.43% and 95.43%, obtained from RGNNV and RG/SJ strains, respectively) (Table 1). If the average values for the three strains are considered, the highest ones were observed in DLB-1 (94.64%, range 92.96–95.43) followed by E-11 (90.97% range 85.73–97.43) and the lowest in SaB-1 cells (18.22%, range 17.71–19.11).

**Figure 1:**
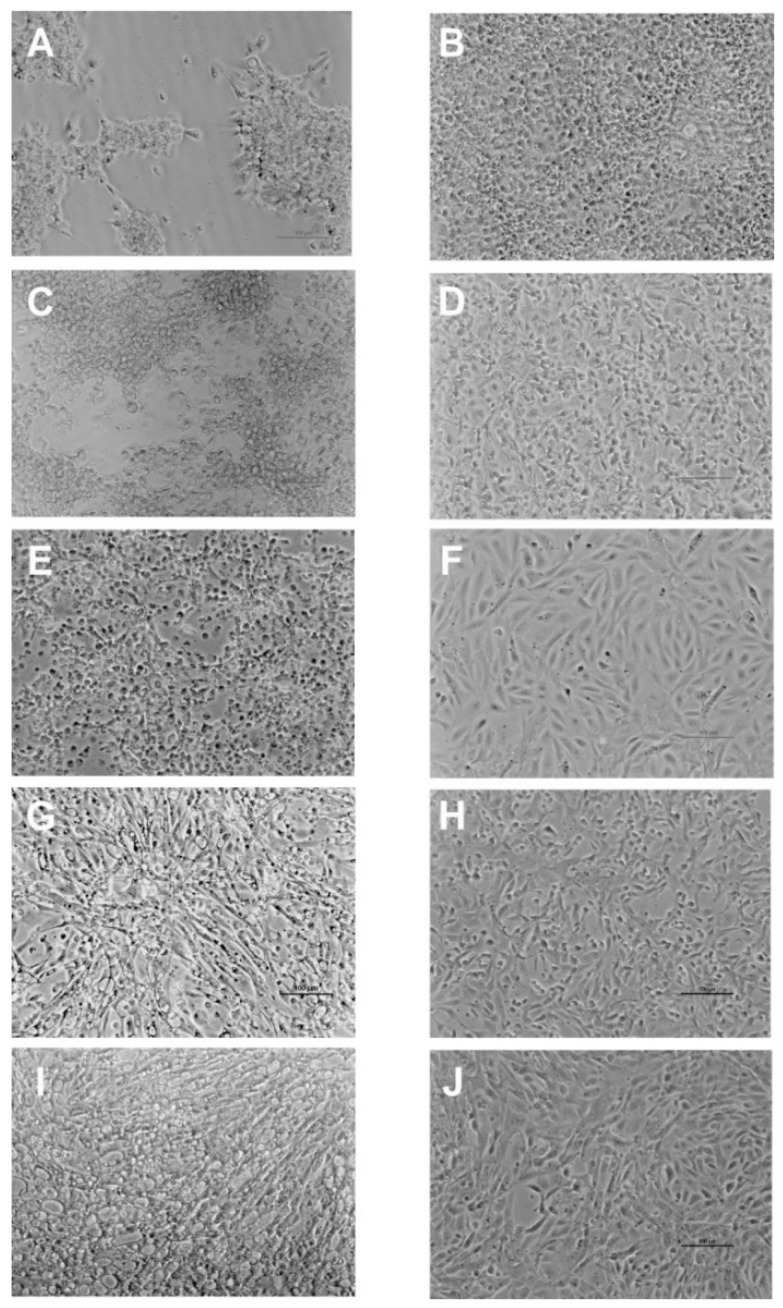
Characteristic cytopathic effect (CPE) developed by RGNNV strain in E-11 (**A**), GF-1 (**C**), SAF-1 (**E**), DLB-1 (**G**) and SaB-1 (**I**) cell lines after 7dpi. Control: Non-inoculated E-11 (**B**), GF-1 (**D**), SAF-1 (**F**), DLB-1 (**H**) and SaB-1 (**J**) cells.

CPE appearance and progression in the different cell lines was monitored daily and compared with non-inoculated cells (Figure 1B,D,F,H,J). Characteristic cell vacuolation was observed in all cell lines, but vacuoles were detected earlier and progressed quicker to monolayer destruction in E-11 cells. At 7 dpi, partial monolayer destruction was observed in both E-11 and GF-1 (Figure 1A,C), whereas in DLB-1, SAF-1, and SaB-1 different CPE progression was seen: in DLB-1 rounded and refractile cells, in SAF-1 cell shrinkage and vacuolation, and in SaB-1 extended vacuolation (Figure 1E,G,I). 

To assess viral production in each cell line, an initial titration was performed in both E-11 and GF-1 cells for comparative purposes (data not shown). For RGNNV and RG/SJ, no significant differences in the titre were observed regardless of the cell used. However, the SJNNV strain titres were around 1 log higher in E-11 than in GF-1. Therefore, all subsequent titrations were performed in E-11. Viral titres obtained from E-11 and GF-1 cell lines were high (in the range of 10^7^–10^8^ TCID_50_/mL), except for the SJNNV strain in GF-1 (1.52 × 10^5^) (Table 1). However, in the other three lines the average TCID_50_ values were lower, 8.26 × 10^4^ in SAF-1 and 1.36 × 10^3^ and 4.28 × 10^2^ in DLB-1 and SaB-1, respectively. Regarding the progeny production of each parental strain (calculated as the production rate = VP/AV) in the different cell lines, E-11 and GF-1 showed good rates for RGNNV and RG/SJ strains (4.9–6.51 × 10^3^ and 2.29–1.59 × 10^3^, respectively) although with lower values for SJNNV (2.75 × 10^2^ and 17.8 in E-11 and GF-1, respectively). In SAF-1 the production rates for RGNNV and RG/SJ strains were 6.1 and 5.8, respectively. However, the SJNNV progeny value was lower than that of the initial inoculum and therefore the production rate was lower than 1 (4.5 × 10^−1^). Likewise, DLB-1 and SaB-1 showed production rates ranging from 5.8 × 10^−2^ to 1.2 × 10^−1^. The relative rate of production (RRP), calculated as the ratio between the production rate of each virus and the lowest value of production, confirmed these observations because the highest values were obtained for the reassortant and RGNNV strains in E-11 and GF-1, whereas the lowest were obtained for the SJNNV strain in DLB-1 and SaB-1. Furthermore, the comparison of the efficacy of viral replication within each cell (RRPC) indicated that the RG/SJ and RGNNV strains were the most effective in all cell lines, but the differences from SJNNV production varied greatly. Thus, whereas the highest difference was observed in GF-1 (RGNNV production was 129-times higher than that of SJNNV), in E-11 the difference was 17.82-fold and in SaB-1 only 2-fold.

### 2.2. Viral Kinetics

For each viral strain, the production of infective particles was measured both intra- and extracellularly. In addition, NNV replication was confirmed with the RNA1 synthesis data up to 24 hpi.

**Figure 2:**
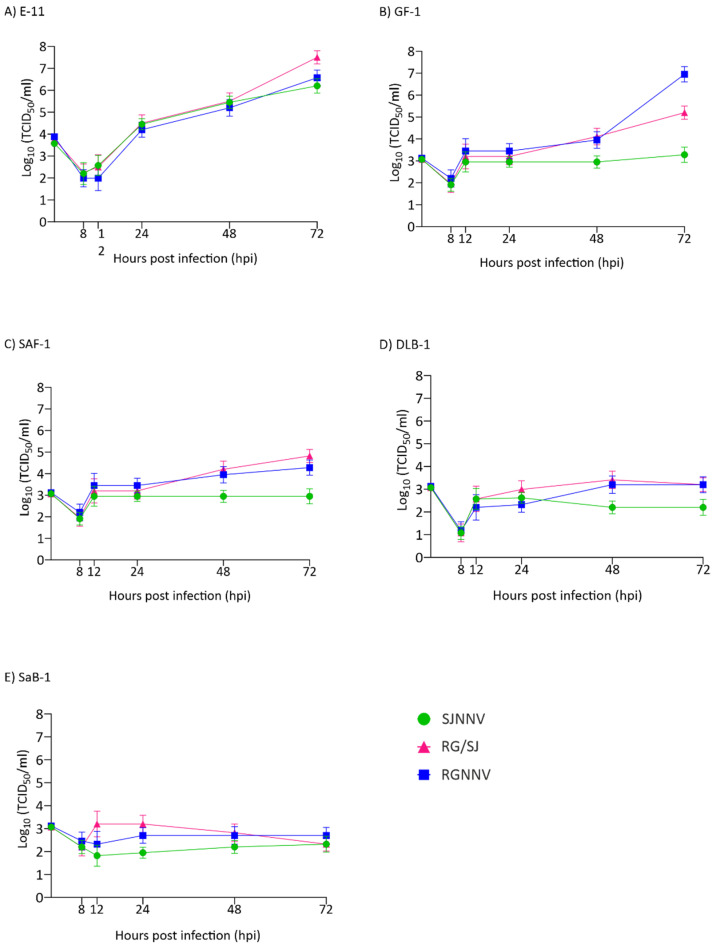
Intracellular replication kinetics. Monolayers of E-11 (**A**), GF-1 (**B**), SAF-1 (**C**), DLB-1 (**D**), and SaB-1 (**E**) cells in 12-well plates were infected with SJNNV, RGNNV, and RG/SJ strains at a MOI of 0.01. The cells were scraped and harvested at 8, 12, 24, 48, and 72 h post infection (hpi). Infectious titres were determined by the end-point titration method in E-11 cells and are expressed as mean log_10_ TCID_50_ ± SD (n = 3) per millilitre.

Regarding intracellular replication, our results showed differences between the three viral strains and between the cell lines (Figure 2). We observed that in the E-11 cell line, replication was exponential from 12 h onwards (Figure 2A). The RG/SJ strain showed the maximum production (3.16 × 10^7^ TCID_50_/mL) followed by RGNNV (3.75 × 10^6^) and SJNNV (1.58 × 10^6^) (Figure 2A). In GF-1, the kinetics were different showing an initial increase of 1–2 logs depending on the strain at 12 hpi, followed by a plateau. From 48 h onwards a sudden increase was observed in RGNNV and RG/SJ strain replication to reach 8.89 × 10^6^ and 1.58 × 10^5^ TCID_50_/mL, respectively (Figure 2B), whereas the SJNNV strain maintained a titre of around 10^3^ until the end of the experiment. Poor productivity was observed in the SAF-1, DLB-1, and SaB-1 cell lines (Figure 2C–E). However, in SAF an increase of 1-1.5 log was detected in RG and RG/SJ final titres. 

**Figure 3:**
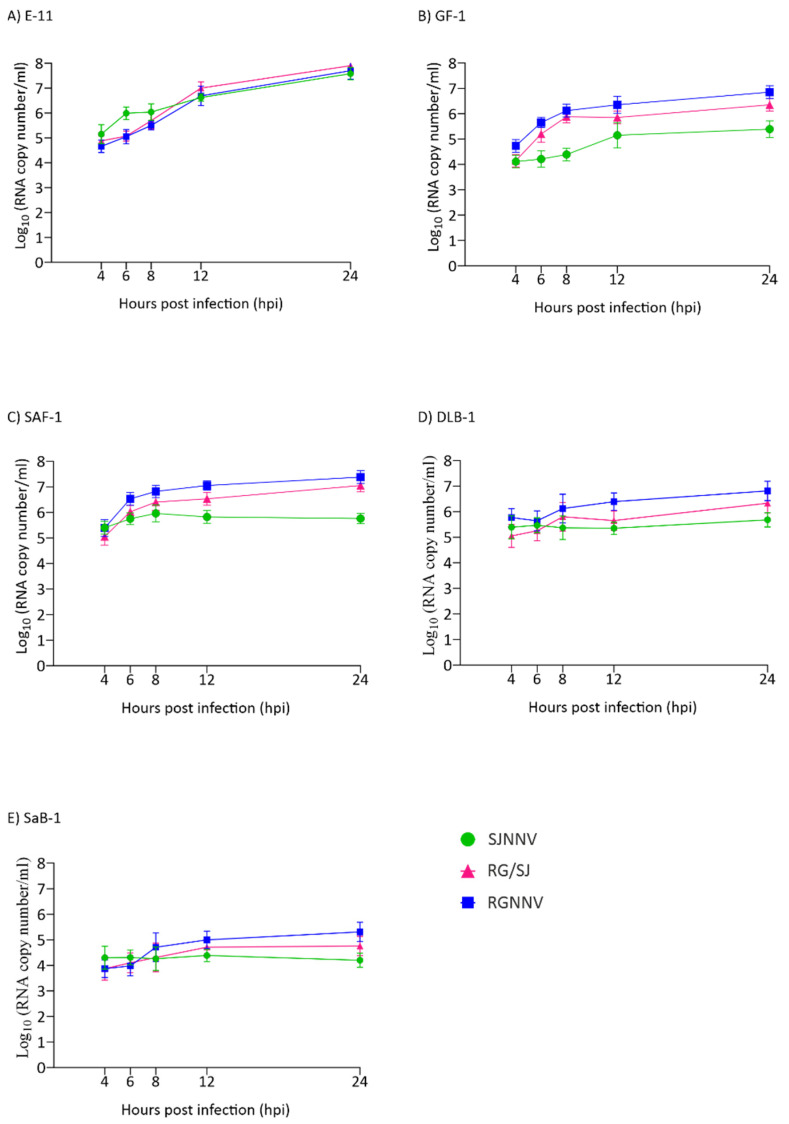
Genome synthesis in cell lysates. A quantitative real-time PCR (qPCR) was used to determine the number of RNA1 copies per millilitre of cell lysates at different time points. Monolayers of E-11 (**A**), GF-1 (**B**), SAF-1 (**C**), DLB-1 (**D**), and SaB-1 (**E**) cells in 12-well plates were infected with SJNNV, RGNNV, and RG/SJ strains at a MOI of 0.01. The cells were scraped off and harvested at 4, 6, 8, 12, and 24 h post infection (hpi). Results are expressed as log_10_ RNA1 copy number ± SD (n = 3) per millilitre.

Because all titrations were performed in E-11 cells and in order to avoid the effect of a poor adaptation of viruses grown in the other cell lines, the synthesis of RNA1 up to 24 hpi was also quantified. As observed with the viral particle production, a clear exponential increase in RNA1 copies was observed in the E-11 cell line (Figure 3A). In GF-1 and SAF-1 a 2-log increase was observed for the RGNNV and RG/SJ strains (Figure 3B,C) and in DLB-1 only for the RGNNV strain (Figure 3D), whereas in SaB-1 the increment was only 1 log (Figure 3E). The SJNNV RNA1 copy number did not grow when the virus was cultured in either SAF-1, DLB-1 or SaB-1.

**Figure 4:**
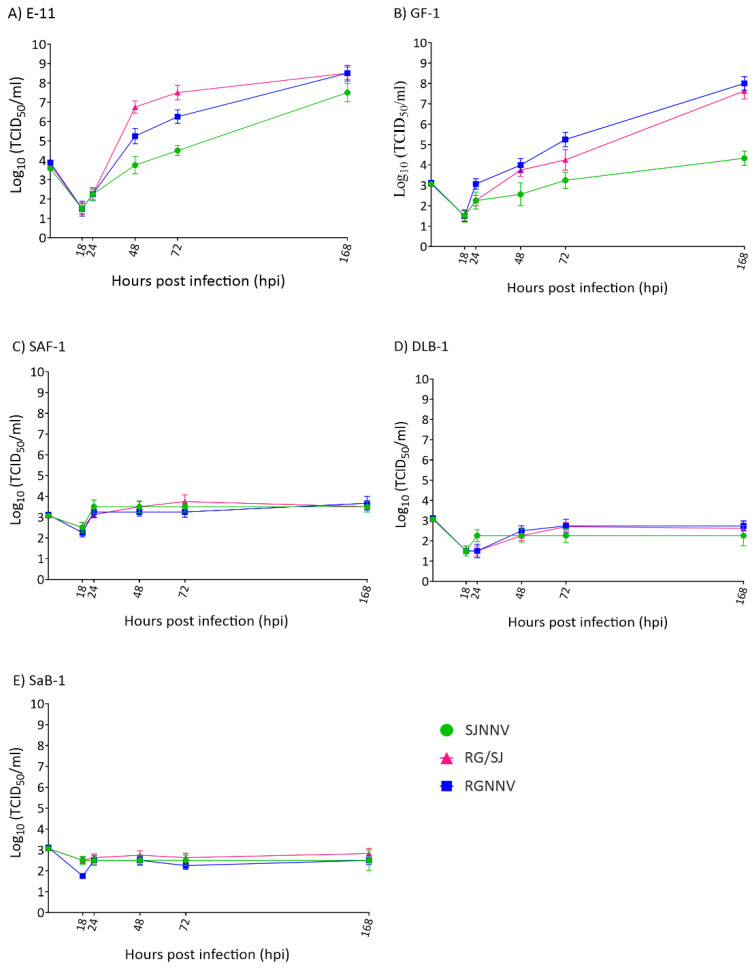
Viral production in cell supernatants. Monolayers of E-11 (**A**), GF-1 (**B**), SAF-1 (**C**), DLB-1 (**D**), and SaB-1 (**E**) cells in 25 cm^2^ flasks were infected with SJNNV, RGNNV, and RG/SJ strains at a MOI of 0.01 and supernatants harvested at the indicated time points. Infectious titres were determined by the end-point titration method in E-11 cells and are expressed as mean log_10_TCID_50_ ± SD (n = 3) per millilitre.

Finally, we studied the progeny production of the three viral strains in each cell line. As observed in the intracellular replication, only supernatants recovered from the E-11 and GF-1 cell lines showed exponentially increased values (Figure 4A,B), whereas no variations in viral titres were recorded in SAF-1, DLB-1, and SaB-1 (Figure 4C–E). As shown in Figure 4A, in E-11 the RG and RG/SJ strains reached identical values (3.16 × 10^8^ TCID_50_/mL) after 168 h /7 d although SJNNV replication was slightly lower (3.2 × 10^7^). In GF-1 cells, again both RG and RG/SJ strains were the most effective (10^8^ and 4.25 × 10^7^, respectively), but the SJ strain reached a clearly lower titre (2.15 × 10^5^) (Figure 4B). In the other three cell lines, viral titres showed only an initial and slight increase (SAF-1 and DLB-1) or remained stable throughout the experiment (SaB-1) displaying final values between 10^2^ and 10^3^.

## 3. Discussion

At present, VER is one of the most threatening viral diseases affecting marine farmed fish worldwide. Isolation and further characterization of NNV strains causing disease in different fish species is essential for a better understanding of the viral agent and for the design of effective control measures. Although in recent years different cell lines have been reported to be susceptible to NNV, most of them have only been tested with strains belonging to the RGNNV genotype. In the present study, we analysed both the susceptibility and the permissiveness of five fish cell lines (E-11, GF-1, SAF-1, DLB-1, and SaB-1) to three NNV strains (one RGNNV, one SJNNV, and one reassortant RG/SJ). 

A cell line is considered susceptible to a given virus when receptors that allow viral attachment are displayed on the cell surface. Therefore, the assessment of the adsorption efficacy is a good measure of cell susceptibility [26,27]. Our AE results indicate that E-11 and DLB-1 (with average values of 90.99 and 94.63%, respectively) are highly susceptible to RGNNV, SJNNV, and RG/SJ strains. SAF-1 also showed high susceptibility (AE values ranging from 81.66 to 98.63%) whereas GF-1 can be regarded as moderately susceptible (AE 49.16–54.04%). On the contrary, SaB-1 can be considered a poorly susceptible cell line (AE values no higher than 19%). To date, different molecules have been identified as putative NNV receptors including sialic acid [28] in SSN-1 cells (and therefore in E-11) and grouper heat shock cognate protein 70 (GHSC70) in GF-1 [29]. Differences in the sialic acid and HSC70 molecule structure and differential interaction with the NNV capsid P domain may account for the differential adsorption efficacy observed in E-11 and GF-1 cells. Another putative NNV receptor, Nectin-4, has recently been identified in primary grouper brain cells [30]. Given that nectins are widely expressed in grouper brain [31], they may also be present in brain cell lines from different fish species, as DLB-1 and SaB-1 [24,25]. However, the different adsorption results obtained in both cell lines seem to point to different receptors.

A permissive cell is one that allows the completion of the viral cycle and the release of viral progeny to the intracellular space ready to infect new cells. Because of that, besides measuring overall viral production after 7 days, we analysed both intracellular and extracellular viral kinetics. E-11 and GF-1 cells provided the highest production rates for RGNNV and RG/SJ (around 10^3^). If permissive cell lines are defined as those producing 100-fold viral increases above the inoculum [32], both cell lines can be regarded as fully permissive for these viral types. The SJNNV production rate was lower in both cells, which could be due to the incubation temperature used, as it has been reported that at 25 °C this strain shows reduced fitness [33]. However, whereas a 200-fold increase was observed in E-11, in GF-1 the production rate was only 17.8, suggesting that factors other than temperature limit SJNNV replication in this cell line. In addition, differences in viral kinetics were observed between both cell lines. Thus, whereas in E-11 the three strains showed exponential replication, in GF-1 exponential RGNNV and RG/SJ intracellular replication was delayed until 48 hpi, and the growth curve of the SJNNV strain plateaued after a slight initial increase, which was confirmed by the extracellular progeny production (10^8^, 10^7^, and 10^4^ TCID_50_/mL for RGNNV, RG/SJ, and SJNNV strains, respectively). On the other hand, DLB-1 and SaB-1 final viral titres obtained after a 7-day incubation were lower than that of the inoculum which led to production rates below 1. These results were confirmed by the analysis of viral kinetics because similar TCID_50_ values were recorded throughout the incubation period when both intracellular and extracellular production were assessed. Regarding SAF-1, although SJNNV showed similar behaviour to that observed in DLB-1 and SaB-1, RGNNV and RG/SJ strains displayed production rates slightly above 1, indicating a very modest viral output. The analysis of the viral kinetics evidenced intracellular production (increase of 1–1.5 logs), but no extracellular production. These results suggest that the release of viral progeny from infected cells is restricted. Cell lysis is a common outcome of viral infection by most non-enveloped viruses and allows the release of viral progeny [34]. Therefore, the non-lytic CPE observed in SAF-1 cells seems to prevent the release of viral progeny produced in the initially infected cells and spread to uninfected cells. 

To confirm the poor intracellular replication in DLB-1 and SaB-1 and to rule out that the low TCID_50_ values could be due to a poor adaptation of the virus produced in these cells to E-11, RNA1 synthesis was quantified. The RNA1 kinetics up to 24 hpi showed that the highest values were obtained in E-11 cells (with a 3-log increase), whereas a 2 log-increase was produced in the RGNNV and RG/SJ strains grown in GF-1 and SAF-1 and in the RGNNV strain in DLB-1. Conversion of the RNA quantification data into TCID_50_ values using a crude virus standard as described by the authors of [35], confirmed that very similar titres (10^2^–10^3^ TCID_50_/mL) would be obtained in GF-1 and SAF-1 with RGNNV and RG/SJ strains and in DLB-1 with RGNNV strain. Since titration values of virus produced in GF-1 were clearly higher than those obtained from the virus grown in SAF-1 and DLB-1, these results suggest that titration in E-11 may have underestimated the replication of RG/SJ and/or RGNNV strains in SAF-1 and DLB-1, respectively. DLB-1 has been previously reported to support NNV replication with higher viral titres than those obtained in this study [36]. As in both studies, titration was performed in E-11 cells, the differences in viral production could be related to the lower MOI used in the present work (0.01 vs. 0.1) and the interferon (IFN) production by DLB infected cells [36]. High MOI values theoretically ensure that all cells are simultaneously infected [37]. Therefore, as MOI increases, less uninfected cells could be protected against NNV infection by the IFN released by the infected cells, suggesting that the IFN pathway is not powerful enough to control viral replication [34]. However, when low MOI values are used the proportion of infected and non-infected cells is reversed and an antiviral state can be induced in the non-infected cells, which would lead to a lower viral production. This phenomenon has already been observed in barramundi brain cells persistently infected with NNV [38] as well as in EPC cells persistently infected with infectious pancreatic necrosis virus, which show antiviral activity against viral haemorrhagic septicaemia virus [39]. Finally, as SSN-1 and E-11 cells are persistently infected with the C-type retrovirus SnRV, it has been suggested that SnRV may play an important role in NNV replication [21]. However, NNV yield in DLB-1 and SaB-1, both containing the same retrovirus [24,25], was clearly lower than that obtained in E-11, suggesting that factors other than the presence of SnRV are involved in the high viral proliferation obtained in E-11 cells.

## 4. Materials and Methods

### 4.1. Cell Lines and NNV Strains 

Five fish cell lines previously described were used in this study: E-11 cell line (a clone of the SSN-1 cell line), GF-1 (grouper fin 1), SAF-1 (*Sparus aurata* fin 1), DLB-1 (*Dicentrarchus labrax* brain 1), and SaB-1 (*Sparus aurata* brain 1). 

The NNV strains used in this study were the following: SGWak97 and SJNag93 belonging to the RGNNV and SJNNV genotypes (RGNNV and SJNNV hereafter, respectively) [20]; and SpSsIAusc160.03, a reassortant RGNNV/SJNNV strain isolated from diseased farmed sole [10], hereafter RG/SJ). The viruses were propagated in semiconfluent E-11 cells with Leibovitz L-15 medium (Lonza, Basilea, Switzerland) containing 5% fetal bovine serum (FBS, Lonza, Basilea, Switzerland) at 25 °C and stored at −80 °C, as previously described [35]. Viral strains were titrated in triplicate by the endpoint dilution method on 96-well plates and expressed as 50% tissue culture infective dose (TCID_50_) according to the method described in [40].

### 4.2. Experimental Design for the Viral Replication Study 

The capacity of the NNV strains to replicate in the different cell lines was assessed by the evaluation of the adsorption and final progeny production. In addition, for a detailed analysis of viral replication in each cell line, intracellular and extracellular viral kinetics were studied. All assays were run in triplicate.

#### 4.2.1. Differential Replication in Five Putative Susceptible Cell Lines

Each strain was inoculated at a multiplicity of infection (MOI) of 0.01 in 12-well plates (Sarstedt, Munich, Germany) containing semi-confluent monolayers of the corresponding cell lines (E-11, GF-1, SAF-1, DLB-1, and SaB-1). After 45 min of adsorption, the remaining inoculum was removed and stored at −20 °C until later use. The monolayers were then washed three times and covered with an L-15 medium, and the plates were incubated at 25 °C and visualized daily for CPE detection. When the cytopathic effect (CPE) was extensive or at 7 days post inoculation (dpi), the supernatants were collected. Those cells lines that showed a slow CPE progression at this time point were subjected to three freeze–thaw cycles. In both cases the cell debris was removed by low-speed centrifugation (3000 rpm for 10 min). At the end of the assay, three sets of titrations were performed: (i) the total inoculated virus (TIV), (ii) the remaining inoculum from each of the inoculated wells (non-attached virus; NAV) and (iii) the viral progeny, PV). Titrations were performed in triplicate in E-11 and GF-1 cells for comparative purposes. The remaining cell lines did not grow properly in 96-well plates and consequently titration was not accomplished.

#### 4.2.2. Kinetics of Viral Production

To study the kinetics of viral production, two time-course experiments were performed. Intracellular production was assessed by infecting each cell line (in 12-well plates) with RGNNV, SJNNV, and RG/SJ strains at a MOI of 0.01. After a 45 min adsorption period, the inoculum was removed, and the monolayers were washed three times, overlaid with fresh medium, and further incubated at 25 °C. After 8, 12, 24, 48, and 72 h post inoculation (hpi), supernatants were discarded, and cells were washed, scraped, and stored at −20 °C for later titration. For the kinetics of extracellular production, cell lines were seeded in 25 cm^2^ flasks (Sarstedt) that were infected as described above. After 18, 24, 48, 72, and 168 hpi, supernatant aliquots were collected and stored at −20 °C until titration. All titrations were performed in triplicate in E-11 cells.

#### 4.2.3. Genome Synthesis within Cells

Each cell line was seeded in 12-well plates that were infected at a MOI of 0.01 with either SGWak97, SJNag93, or Ss160 strains. Following a 45 min adsorption period, the inoculum was removed, and the monolayers were washed three times, overlaid with fresh L-15 medium and further incubated at 25 °C. After 4, 6, 8, 12, and 24 h of in vitro infection, supernatants were discarded, cells were washed, scraped, and stored at −20 °C for RT-qPCR quantification.

The extraction of total RNA was carried out using the Ezna Total RNA purification kit (VWR, Radnor, PA, USA) following the indications of the manufacturer. The synthesis of cDNA was performed using Superscript IV RT (ThermoFisher Scientific, Waltham, MA, USA) with random primers (ThermoFisher Scientific, Waltham, MA, USA). The qPCR reactions were performed with 2 μL of cDNA samples in a final volume of 20 μL, using iQTM-SYBRGreenSupermix (Bio-Rad, Hercules, CA, USA) and 200 nM of primers SnodR1 F/R [41] in a CFX96TM Real-Time PCR Detection System (Bio-Rad, Hercules, CA, USA) as previously described [42]. All samples were tested in triplicate. A 10-fold dilution series containing 10^7^–10^1^ copies of a plasmid DNA containing the full-length cDNA sequence of SpSsIAusc160.03 RNA1 was used to create a standard curve.

### 4.3. Calculus and Statistical Analysis

As previously indicated, the data correspond to the mean value from 3 independent replicas. The attached virus (AV) was calculated according to the following formula AV = VI − NAV, where VI is the total inoculated virus and NAV, the non-attached virus. Adsorption efficacy (AE) as a percentage was calculated from the ratio between the attached virus and viral inoculum (AE = AV/IV × 100). The production rate or the number of viruses produced in the progeny per each parental virus was calculated as the ratio between viral progeny and attached virus (VP/AV). The relative ratio of production (RRP) for all cell lines was calculated as the ratio between the production rate of each virus and the lowest value of production rate. The relative ratio of production in each cell line (RRPC) was calculated as the ratio between the production rate of each virus and the lowest value of production rate observed in that cell line. 

Statistical analyses were carried out using GraphPad Prism version 6.00 (GraphPad Software, La Jolla, CA, USA). Viral quantification data were subjected to a two-way ANOVA followed by Tukey’s multiple comparisons test.

## 5. Conclusions

In this study we compared the capacity of five fish cell lines to support the NNV cycle. Our findings highlight the need to assess both viral attachment, which would be indicative of cell line susceptibility, and replication, indicative of cell permissiveness, and to use strains belonging to different genotypes. Cell susceptibility analysis showed differences between strains although with high adsorption efficacy and only SaB-1 can be considered poorly susceptible. However, whereas the three NNV strains replicated well in E-11, SJNNV growth was clearly lower and slower in GF-1. Intracellular replication was observed in SAF-1, but viral progeny is not released to the extracellular environment. Finally, although low titration values were obtained in E-11 from virus grown in SAF-1, DLB-1, and SaB-1, genome synthesis suggests an underestimation of RGNNV replication in the two first cell lines. Based on all these results, E-11 seems to be the most appropriate cell for in vitro culture of RGNNV, SJNNV, and reassortant strains. 

## Figures and Tables

**Table 1:** NNV differential replication in the five fish cell lines tested.

		Viral Inoculum (VI)		Attached Virus (AV)			Viral Progeny (VP)				
Cell Line	Strain	TCID_50_/mL	Log ± Sdev	TCID_50_/mL	Log ± Sdev	AE	TCID_50_/mL	Log ± Sdev	Prod Rate	RRP	RRPC
E-11	RGNNV	2.81 × 10^4^	4.45 ± 0.02	2.74 × 10^4^	4.44 ± 0.00	97.43%	1.34 × 10^8^	8.01 ± 0.38	4.91 × 10^3^	84,263.25	17.82
	SJNNV	1.73 × 10^4^	4.24 ± 00	1.49 × 10^4^	4.17 ± 0.04	85.73%	4.1 × 10^6^	6.60 ± 0.14	2.75 × 10^2^	4728.57	1
	RG/SJ	1.84 × 10^4^	4.27 ± 0.02	1.65 × 10^4^	4.22 ± 0.02	89.73%	1.08 × 10^8^	8.01 ± 0.14	6.51 × 10^3^	111,689.61	23.62
GF-1	RGNNV	2.81 × 10^4^	4.45 ± 0.02	1.52 × 10^4^	4.18 ± 0.28	54.04%	3.48 × 10^7^	7.43 ± 0.43	2.29 × 10^3^	39,363.62	128.84
	SJNNV	1.73 × 10^4^	4.24 ± 0.19	8.53 × 10^3^	3.91 ± 0.18	49.16%	1.52 × 10^5^	5.18 ± 0.00	17. 8	305.53	1
	RG/SJ	1.84 × 10^4^	4.27 ± 0.02	9.57 × 10^3^	3.96 ± 0.17	51.98%	1.52 × 10^7^	7.18 ± 0.00	1.59 × 10^3^	27,224.88	89.11
SAF-1	RGNNV	2.81 × 10^4^	4.45 ± 0.19	2.42 × 10^4^	4.38 ± 0.00	86.0%	1.47 × 10^5^	5.10 ± 0.29	6.1	104.27	13
	SJNNV	2.88 × 10^4^	4.46 ± 0.26	2.84 × 10^4^	4.45 ± 0.00	98.63%	1.3 × 10^4^	4.10 ± 0.14	4.5 × 10^−1^	7.83	1
	RG/SJ	1.84 × 10^4^	4.27 ± 0.18	1.50 × 10^4^	4.18 ± 0.03	81.66%	8.8 × 10^4^	4.85 ± 0.38	5.8	100.52	12.83
DLB-1	RGNNV	1.24 × 10^4^	4.09 ± 0.14	1.18 × 10^4^	4.07 ± 0.00	95.16%	2.31 × 10^3^	3.35 ± 0.14	1.95 × 10^−1^	3.34	3.34
	SJNNV	2.88 × 10^4^	5.46 ± 0.04	2.69 × 10^4^	5.43 ± 0.01	93.32%	1.57 × 10^3^	3.1 ± 0.29	5.8 × 10^−2^	1.00	1
	RG/SJ	1.97 × 10^4^	5.81 ± 0.24	1.88 × 10^4^	5.80 ± 0.01	95.43%	1.91 × 10^3^	3.26 ± 0.25	1 × 10^−1^	1.75	2
SaB-1	RGNNV	1.58 × 10^4^	4.20 ± 0.25	2.81 × 10^3^	3.45 ± 0.049	17.71%	3.52 × 10^2^	2.5 ± 0.25	1.2 × 10^−1^	2.16	2
	SJNNV	2.81 × 10^4^	4.45 ± 0.09	4.98 × 10^3^	3.70 ± 0.01	17.71%	3.52 × 10^2^	2.5 ± 0.25	7.07 × 10^−2^	1.21	1
	RG/SJ	2.81 × 10^4^	4.45 ± 0.1	5.37 × 10^3^	3.73 ± 0.00	19.11%	8.54 × 10^2^	2.92 ± 0.14	1.59 × 10^−1^	2.73	2.25

The attached virus (AV) was calculated as the difference between the viral inoculum and non-attached virus; the adsorption efficacy (AE) was calculated from the ratio between attached virus and viral inoculum as follows AE = AV/VI × 100; the production rate per each parental virus was calculated as the ratio between the viral progeny and the attached virus (VP/AV); when no cell destruction was observed cells were subjected to three freeze-thaw cycles; and the relative rate of production (RRP) for all cell lines was calculated as the ratio between the production rate of each virus and the lowest value of the production rate. The production rate per cell was calculated as the ratio between the production rate of each virus and the lowest value obtained in each cell line. All titrations were performed in E-11 cells. Results from each cell line have been coloured differently to facilitate comparison.

## Data Availability

The authors confirm that the data supporting the finding of this study are available within the article. Raw data are available from the corresponding author (I.B.), upon reasonable request.

## Abbreviations

NNV: Nervous necrosis virus; VER: viral encephalopathy and retinopathy; VNN: viral nervous necrosis. BFNNV: barfin flounder nervous necrosis virus; RGNNV: redspotted grouper nervous necrosis virus; SJNNV: striped jack nervous necrosis virus; TPNNV: tiger puffer nervous necrosis virus; SSN-1: striped snakehead cells; E-11: Clon of SSN-1 cells; GF-1: grouper fin cells; SAF-1: *Sparus aurata* fin cells; DLB-1: *Dicenthrarchus labrax* brain cells; SaB-1: *Sparus aurata* brain cells; MOI: multiplicity of infection; hpi: hours post-inoculation; CPE: cytopathic effect; AV: attached virus; VI: total inoculated virus; NAV: non-attached virus; AE: adsorption efficacy; RRP: relative ratio of production; RRPC: relative ratio of production in each cell line.

## References

[1] Bandín I., Souto S. (2020). Betanodavirus and VER Disease: A 30-year Research Review. Pathogens.

[2] Mori K.-I., Nakai T., Muroga K., Arimoto M., Mushiake K., Furusawa I. (1992). Properties of a new virus belonging to nodaviridae found in larval striped jack (*Pseudocaranx dentex*) with nervous necrosis. Virology.

[3] Chen L.J., Su Y.C., Hong J.R. (2009). Betanodavirus non-structural protein B1: A novel anti-necrotic death factor that modulates cell death in early replication cycle in fish cells. Virology.

[4] Fenner B.J., Thiagarajan R., Chua H.K., Kwang J. (2006). Betanodavirus B2 is an RNA interference antagonist that facilitates intracellular viral RNA accumulation. J. Virol..

[5] Su Y.C., Wu J.L., Hong J.R. (2009). Betanodavirus non-structural protein B2: A novel necrotic death factor that induces mitochondria-mediated cell death in fish cells. Virology.

[6] Iwamoto T., Mise K., Takeda A., Okinaka Y., Mori K.I., Arimoto M., Okuno T., Nakai T. (2005). Characterization of striped jack nervous necrosis virus subgenomic RNA3 and biological activities of its encoded protein B2. J. Gen. Virol..

[7] Mézeth K.B., Patel S., Henriksen H., Szilvay A.M., Nerland U.H. (2009). B2 protein from betanodavirus is expressed in recently infected but not in chronically infected fish. Dis. Aquat. Organ..

[8] Sommerset I., Nerland A. (2004). Complete sequence of RNA1 and subgenomic RNA3 of Atlantic halibut nodavirus (AHNV). Dis. Aquat. Organ..

[9] Nishizawa T., Furuhashi M., Nagai T., Nakai T., Muroga K. (1997). Genomic classification of fish nodaviruses by molecular phylogenetic analysis of the coat protein gene. Appl. Environ. Microbiol..

[10] Olveira J.G., Souto S., Dopazo C.P., Thiéry R., Barja J.L., Bandín I. (2009). Comparative analysis of both genomic segments of betanodaviruses isolated from epizootic outbreaks in farmed fish species provides evidence for genetic reassortment. J. Gen. Virol..

[11] Toffan A., Pascoli F., Pretto T., Panzarin V., Abbadi M., Buratin A., Quartesan R., Gijon D., Padros F. (2017). Viral nervous necrosis in gilthead sea bream (*Sparus aurata*) caused by reassortant betanodavirus RGNNV/SJNNV: An emerging threat for Mediterranean aquaculture. Sci. Rep..

[12] Volpe E., Gustinelli A., Caffara M., Errani F., Quaglio F., Fioravanti M.L., Ciulli S. (2020). Viral nervous necrosis outbreaks caused by the RGNNV/SJNNV reassortant betanodavirus in gilthead sea bream (*Sparus aurata*) and European sea bass (*Dicentrarchus labrax*). Aquaculture.

[13] Yoshikoshi K., Inoue K. (1990). Viral nervous necrosis in hatchery-reared larvae and juveniles of Japanese parrotfish, *Oplegnathus fasciatus* (Temminck & Schlegel). J. Fish Dis..

[14] Breuil G., Bonami J.R., Pepin J.F., Pichot Y. (1991). Viral infection (picorna-like virus) associated with mass mortalities in hatchery-reared sea-bass (*Dicentrarchus labrax*) larvae and juveniles. Aquaculture.

[15] Mori K., Nakai T., Nagahara M., Muroga K., Mekuchi T., Kanno T. (1991). A viral disease in hatchery-reared larvae and juveniles of redspotted grouper. Fish Pathol..

[16] Munday B.L., Langdon J.S., Hyatt A., Humphrey J.D. (1992). Mass mortality associated with a viral-induced vacuolating encephalop- athy and retinopathy of larval and juvenile barramundi, Lates calcarifer Bloch. Aquaculture.

[17] Nguyen H.D., Mekuchi T., Imura K., Nakai T., Nishizawa T., Muroga K. (1994). Occurrence of viral nervous necrosis (VNN) in hatchery-reared juvenile Japanese flounder *Paralichthys olivaceus*. Fish. Sci..

[18] Grotmol S., Totland G.K., Kvellestad A., Fjell K., Olsen A.B. (1995). Mass mortality of larval and juvenile hatchery-reared halibut (*Hippoglossus hippoglossus* L.) associated with the presence of virus-like particles in vacuolated lesions in the central nervous system and retina. Bull Eur. Assoc. Fish Pathol..

[19] Frerichs G.N., Rodger H.D., Peric Z. (1996). Cell culture isolation of piscine neuropathy nodavirus from juvenile sea bass, *Dicentrarchus labrax*. J. Gen. Virol..

[20] Iwamoto T., Mori K., Arimoto M., Nakai T. (1999). High permissivity of the fish cell line SSN-1 for piscine nodaviruses. Dis. Aquat. Organ..

[21] Iwamoto T., Nakai T., Mori K., Arimoto M., Furusawa I. (2000). Cloning of the fish cell line SSN-1 for piscine nodaviruses. Dis. Aquat. Organ..

[22] Chi S.C., Hu W.W., Lo B.J. (1999). Establishment and characterization of a continuous cell line (GF-1) derived from grouper, *Epinephelus coioides* (Hamilton): A cell line susceptible to grouper nervous necrosis virus (GNNV). J. Fish Dis..

[23] Béjar J., Porta J., Borrego J., Alvarez M. (2005). The piscine SAF-1 cell line: Genetic stability and labeling. Mar. Biotechnol..

[24] Morcillo P., Chaves-Pozo E., Meseguer J., Esteban M.Á., Cuesta A. (2017). Establishment of a new teleost brain cell line (DLB-1) from the European sea bass and its use to study metal toxicology. Toxicol. Vitr..

[25] Ruiz-Palacios M., Esteban M.Á., Cuesta A. (2020). Establishment of a brain cell line (SaB-1) from gilthead seabream and its application to fish virology. Fish Shellfish Immunol..

[26] Lago M., Bandín I., Olveira J.G., Dopazo C.P. (2017). In vitro reassortment between Infectious Pancreatic Necrosis Virus (IPNV) strains: The mechanisms involved and its effect on virulence. Virology.

[27] López-Vázquez C., Bandín I., Panzarin V., Toffan A., Cuenca A., Olesen N.J., Dopazo C.P. (2020). Steps of the replication cycle of the viral haemorrhagic septicaemia virus (VHSV) affecting its virulence on fish. Animals.

[28] Liu W., Hsu C.-H., Hong Y.-R., Wu S.-C., Wang C.-H., Wu Y.-M., Chao C.-B., Lin C.-S. (2005). Early endocytosis pathways in SSN-1 cells infected by dragon grouper nervous necrosis virus. J. Gen. Virol..

[29] Chang J.-S., Chi S.-C. (2015). GHSC70 Is Involved in the Cellular Entry of Nervous Necrosis Virus. J. Virol..

[30] Krishnan R., Qadiri S.S.N., Oh M.-J. (2019). Functional characterization of seven-band grouper immunoglobulin like cell adhesion molecule, Nectin4 as a cellular receptor for nervous necrosis virus. Fish Shellfish Immunol..

[31] Krishnan R., Kim J.-O., Kim J.-O., Qadiri S.S.N., Kim S.-J., Oh M.-J. (2019). Immunoglobulin-like cell adhesion molecules, nectins—Characterization, functional prediction and expression profiling from seven-band grouper, *Hyporthodus septemfasciatus*. Aquaculture.

[32] Dai S., Wu Q., Wu X., Peng C., Liu J., Tang S., Zhang T., Deng F., Shen S. (2021). Differential Cell Line Susceptibility to Crimean-Congo Hemorrhagic Fever Virus. Front. Cell. Infect. Microbiol..

[33] Panzarin V., Cappellozza E., Mancin M., Milani A., Toffan A., Terregino C., Cattoli G. (2014). In vitro study of the replication capacity of the RGNNV and the SJNNV betanodavirus genotypes and their natural reassortants in response to temperature. Vet. Res..

[34] Flint S., Enquist L., Krug R., Racaniello V., Skalka A. (2015). Principles of Virology. Molecular Biology, Pathogenesis, and Control.

[35] Olveira J.G., Souto S., Bandín I., Dopazo C.P. (2021). Development and validation of a SYBR green real time pcr protocol for detection and quantification of nervous necrosis virus (NNV) using different standards. Animals.

[36] Chaves-Pozo E., Bandín I., Olveira J.G., Esteve-Codina A., Gómez-Garrido J., Dabad M., Alioto T., Ángeles Esteban M., Cuesta A. (2019). European sea bass brain DLB-1 cell line is susceptible to nodavirus: A transcriptomic study. Fish Shellfish Immunol..

[37] Shabram P., Aguilar-Cordova E. (2000). Multiplicity of infection/multiplicity of confusion. Mol. Ther..

[38] Wu Y.C.C., Chi S.C.C. (2006). Persistence of betanodavirus in Barramundi brain (BB) cell line involves the induction of Interferon response. Fish Shellfish Immunol..

[39] Jurado M., García-Valtanen P., Estepa A., Perez L. (2013). Antiviral activity produced by an IPNV-carrier EPC cell culture confers resistance to VHSV infection. Vet. Microbiol..

[40] Reed L., Müench H. (1938). A simple method of estimating fifty per cent endpoints. Am. J. Epidemiol..

[41] Olveira J.G., Souto S., Dopazo C.P., Bandín I. (2013). Isolation of betanodavirus from farmed turbot *Psetta maxima* showing no signs of viral encephalopathy and retinopathy. Aquaculture.

[42] Souto S., Olveira J.G., Dopazo C.P., Borrego J.J., Bandín I. (2018). Modification of betanodavirus virulence by substitutions in the 3′ terminal region of RNA2. J. Gen. Virol..

